# F112. LESS SYMPTOMS IN SCHIZOPHRENIA – A RISK FACTOR FOR IMPAIRED INSIGHT OF FUNCTIONING?

**DOI:** 10.1093/schbul/sby017.643

**Published:** 2018-04-01

**Authors:** Olsson Anna-Karin, Maivor Olsson-Tall, Hawar Moradi, Fredrik Hjärthag, Lars Helldin

**Affiliations:** 1 Karlstad University; 2 NU Health Care Hospital; 3 Health Care Hospital, and Karlstad University

## Abstract

**Background:**

People with schizophrenia demonstrate deficits in insight and the ability to self-evaluate their functioning. Research about patients’ ability to recognize their psychotic symptoms is well established, but recent findings show that there are still unexplored fields regarding how patients perceive their level of functioning A previous study showed that patients who overestimate their functioning, also consistently get high scores in interview-based assessment regarding real-world functional performance. The possible consequences of patients’ ability to correctly estimate their function need to be further investigated. The aim of the present study was to examine how the perception of one’s own capacity relate to symptoms in patients with schizophrenia spectrum disorders.

**Methods:**

Data collection took place within the ongoing project Clinical Long-term Investigation of Psychosis in Sweden (CLIPS), which examines psychiatric outpatients. In this study, 222 patients with schizophrenia participated. They were divided into four groups based on their results on the UPSA-B and their self-perceived function; two groups with ordinary function (accurate estimators and under -estimators) and two groups with low function (accurate estimators and over-estimators). The groups were compared regarding psychiatric symptoms, examined using the Positive and Negative Syndrome Scale (PANSS). Non-parametric statistics were used to analyze differences in their symptoms.

**Results:**

There were statistically significant differences in the total score of PANSS across the four groups of function. The following analyses showed significant differences in the negative and general domain. Results from the post hoc examination revealed identical patterns in these two symptom domains. The group with Low function accurate estimators have significantly more severe symptoms compared to the other three groups.

**Discussion:**

The result in the present study showed that patients with low function who overestimate their function have less or the same level of symptoms as patients in the two groups with ordinary functioning. In further studies it is important to investigate if this actually is a result of lower symptom level or if it is due to the impaired insight. This is important since the result in the present study mirror previous results where patients who overestimate a low function also, by clinicians, will be perceived as patients with a higher capacity and less difficulties.

